# Duration of Expulsive Efforts and Risk of Postpartum Hemorrhage in Nulliparous Women: A Population-Based Study

**DOI:** 10.1371/journal.pone.0142171

**Published:** 2015-11-10

**Authors:** Marie-Danielle Dionne, Catherine Deneux-Tharaux, Corinne Dupont, Olga Basso, René-Charles Rudigoz, Marie-Hélène Bouvier-Colle, Camille Le Ray

**Affiliations:** 1 Inserm UMR 1153, Obstetrical, Perinatal and Pediatric Epidemiology Research Team (Epopé), Center for Epidemiology and Statistics Sorbonne Paris Cité, DHU Risks in pregnancy, Paris Descartes University, Paris, France; 2 Department of Obstetrics and Gynecology, McGill University, Montreal, Canada; 3 Montreal University Health Center, Montreal, Canada; 4 Aurore perinatal network, Hôpital de la Croix Rousse, Hospices Civils de Lyon, EA 4129 Université Lyon1, Lyon, France; 5 Department of Epidemiology, Biostatistics and Occupational Health, McGill University, Montreal, Canada; 6 Port Royal Maternity, Cochin-Broca-Hôtel Dieu Hospital, Assistance Publique Hôpitaux de Paris, Paris Descartes University, Paris, France; Shanghai 1st Maternity and Infant hospital of Tongji University, CHINA

## Abstract

**Objective:**

To assess the specific association between the duration of expulsive efforts and the risk of postpartum hemorrhage.

**Methods:**

Population-based cohort-nested case-control study of nulliparous women delivering vaginally in 106 French maternity units between December 2004 and November 2006, including 3,852 women with PPH (blood loss ≥ 500 mL and/or peripartum Hb decrease ≥ 2 g/dL), 1,048 of them severe (peripartum Hb decrease ≥ 4 g/dL or transfusion of ≥ 2 units of red blood cells), and 762 controls from a representative sample of deliveries without hemorrhage in the same population. The association between duration of expulsive efforts and postpartum hemorrhage was estimated by multilevel logistic regression models adjusted for individual and hospital characteristics.

**Results:**

Median duration of expulsive efforts was 18 minutes among controls, 20 minutes among postpartum hemorrhage and 23 minutes among severe postpartum hemorrhage (p<0.01). Duration of expulsive efforts was significantly, positively, and linearly associated with both postpartum hemorrhage and severe postpartum hemorrhage. After adjustment for other risk factors, every additional 10 minutes of expulsive efforts was associated with about a 10% increase in the risk of postpartum hemorrhage (aOR = 1.11 [1.02–1.21]) and severe postpartum hemorrhage (aOR = 1.14 [1.03–1.27]). Oxytocin during labor, duration of active phase of labor, forceps use, episiotomy, perineal tears, and birth weight were also independently associated with both risks.

**Conclusion:**

Duration of expulsive efforts was independently associated with postpartum hemorrhage and severe postpartum hemorrhage. Interventions to shorten the duration of this stage, such as oxytocin, forceps, and episiotomy, are also associated with higher risks of postpartum hemorrhage. Beyond duration, other aspects of the management of active second stage should be evaluated as some might allow it to last longer with a minimal increase in postpartum hemorrhage risk.

## Introduction

Despite improvements in practices and research efforts, postpartum hemorrhage (PPH) remains an important cause of severe maternal morbidity in high-resource countries; severe PPH occurs in 0.5 to 1% of deliveries[1–4]. An increase in their incidence, especially those due to uterine atony, has been recently reported[5–8]. Women's individual characteristics do not appear to explain these observations[9, 10]. It is therefore important to improve our understanding of the risk factors for PPH, in particular, those linked to obstetrical practices.

The duration of the second stage of labor is a reported risk factor for PPH[11–13]. The second stage of labor begins at full dilatation, but the exact instant it occurs (i.e., reaches 10 cm) is difficult to assess. For this reason, the duration of this stage is often underestimated. On the other hand, the duration of expulsive efforts (DEE) is well defined, but depends of obstetrical practices. In the United States, recommendations are at least 2 hours of pushing in multiparous women and at least 3 hours in nulliparous women, before diagnosing arrest of labor[14]. In contrast, a French recommendation, graded C (i.e. low evidence-based), limits the DEE for neonates concerns and recommends to consider an obstetrical intervention if delivery is not imminent after 30 minutes of expulsive efforts[15, 16]. Most studies about consequences of the second stage do not, however, differentiate between its passive phase (i.e. before expulsive efforts) and its active phase, the latter corresponding to the specific stage of expulsive efforts. The greater intrauterine pressure reported during expulsive efforts may be at the root of the increase in the risk of uterine atony and, consequently, of the increase in the risk of PPH[17].

Only two reports have specifically examined PPH as a function of DEE; both found a significant association[18, 19]. These two publications, one from a Canadian randomized trial and the second from a French cohort, were secondary analyses of databases. Furthermore, limited data were available about some important confounding factors, including duration of labor and use of oxytocin, limiting interpretation of their results.

The objective of our study was to assess whether DEE was independently associated with PPH in a large population-based study specifically designed to assess PPH and taken potential risk factors of PPH into account.

## Material and Methods

This study is a population-based nested case-control. The source population is made up by women included in the PITHAGORE6 cluster-randomized controlled trial which took place in 106 French maternity units belonging to six perinatal networks from December 2004 through November 2006, a period covering approximately 147,000 deliveries[3]. These maternity units account for 17% of French maternity units and 20% of deliveries in France. The specific purpose of this trial was to assess the effect of a multifaceted educational intervention to improve the general management of PPH on the incidence of severe PPH. During the study period, all deliveries with PPH were identified: 9,365 women. Cases were identified either based on clinical judgment (blood loss ≥ 500 mL), or by laboratory testing (peripartum hemoglobin decrease ≥ 2 g/dL)[20]. Prepartum haemoglobin was collected as part of routine prenatal care during the last weeks of pregnancy; postpartum haemoglobin was the lowest haemoglobin level found in the three days after delivery. A sample of 1/60 of all deliveries without a hemorrhage in the same maternity units over the same time period (2,412 women) was randomly selected. Detailed information was collected for all of them about management of the hemorrhage, characteristics of the pregnancy and the women, and the course of labor and delivery, including in particular the DEE. As there were no differences between the two arms of the trials, all participating centers are included here. The PITHAGORE6 trial was approved by the Sud Est III Institutional Review Board and the French Data Protection Agency (CNIL).

**Fig 1:**
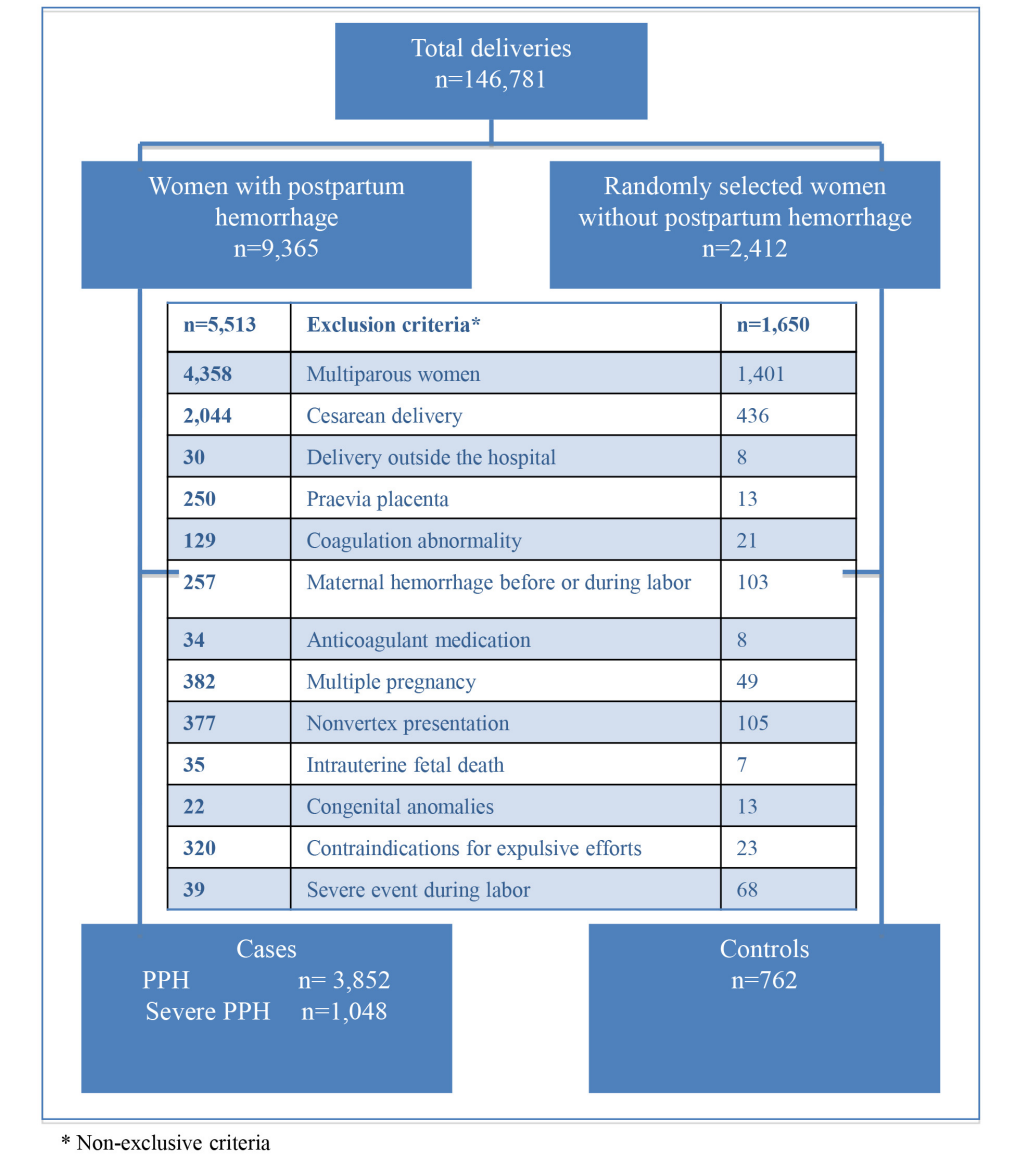
Flow chart. Characteristics of the women, of labor, of delivery and birth weight of the baby were collected from the medical file. Characteristics of the maternity units were collected from a specific questionnaire.

In this study, we restricted the analysis to nulliparas having a vaginal delivery. Women who gave birth outside of the hospital (DEE unavailable) were excluded, as well as specific subgroups at higher risk of PPH (multiple pregnancy, placental abnormalities, congenital or acquired coagulation disorders, recent treatment by anticoagulants or platelet aggregation inhibitors, or a diagnosis of hemorrhage before or during labor, intrauterine fetal death, fetuses with a congenital malformation), and women with a contraindication to expulsive effort (cardiac disease or malformation, neurological malformation or paralysis or neuromuscular disease, general anesthesia before birth, state of shock before or during labor, history of pneumothorax, or severe myopia) and women with non-vertex presentation. For the case-control analysis, two groups of cases were defined, according to PPH severity: 1) all women with PPH (n = 3,852), and 2) women with severe PPH, defined by at least one of the following criteria: transfusion of at least 2 units of packed red blood cells or a fall in the peripartum hemoglobin level of at least 4 g/dL (equivalent to a blood loss of 1000 mL)(n = 1,048). Controls were women from the sample who had not had a PPH (n = 762) (Fig 1).

The exposure of interest was duration of expulsive efforts (DEE). Expulsive efforts started when woman was instructed to push by the midwife or the obstetrician. We examined it both as a continuous and as a categorical variable. To define DEE categories, we used percentiles of DEE among controls (≤50th,] 50–75^th^],]75–90^th^] and >90^th^ percentile). Thus, we created a DEE variable with 4 categories: ≤18 minutes,] 18–27],] 27–37] and >37 minutes).

Duration of previous phases of labor is a major potential confounding factor in the association between DEE and PPH. We defined the duration of previous phases of labor, as the duration between a dilatation of 3 cm and the beginning of expulsive efforts. The specific duration between full dilatation and the beginning of expulsive efforts was not available. To prevent bias from the truncation for the duration of previous phases of labor, we chose to model it as follows. For women admitted with a cervical dilatation of 3 cm or less, the duration of previous phases was the time recorded between 3cm and full dilatation. For women admitted with cervical dilatation >3cm (9% of cases and 16% of controls), we hypothesized a constant speed of cervical dilatation, and the total duration of previous phases of labor was estimated from each woman’s mean cervical dilatation rate (derived from the time of dilatation measurement at admission to the beginning of expulsive efforts)[21].

The distribution of the following characteristics was compared between case and control women in bivariate analyses (chi-2, Fisher exact, Student t, or Wilcoxon tests as appropriate): maternal age (in 3 categories, ≤25, 26–36, >35 years), body mass index (BMI) before pregnancy)(in 3 categories, ≤25, 26–30, >30 kg/m^2^), gestational age at delivery (in 3 categories, <37, 37–40, ≥41 gestation weeks), labor induction (yes/no), epidural analgesia (yes/no), duration of previous phases of labor (continuous variable, hours), total oxytocin dose during labor (continuous variable, IU), mode of delivery (in 3 categories, spontaneous, forceps, vacuum), episiotomy (yes/no), perineal tears (yes/no), birth weight of the baby (in 4 categories, ≤ 3000, 3001–3500, 3501–4000, >4000grams) and prophylactic administration of oxytocin after delivery (yes/no); characteristics of the maternity units: annual number of deliveries (≤ 1500, 1500–3000, > 3000), level of care (1, 2, 3), status (public universitary, public non-universitary, private), randomization group in the Pithagore6 trial (intervention yes/no).

The linearity of the association between the risk of PPH and DEE, and between the risk of PPH and the other continuous quantitative variables (duration of previous phases of labor, and total oxytocin dose during labor) was tested using fractional polynoma. The linear model was valid for DEE, and the duration of previous phases of labor. For the total oxytocin dose, the linear model was rejected; this variable was included in the final models as a categorical variable with categories corresponding to quartiles in the control group (0, >0–0.57 IU, 0.58–1.68, >1.68), since this presentation appeared more meaningful in a clinical perspective; however, models including the dose of oxytocin with the most adequate fractional polynom were also tested in order to check that this did not affect the association between PPH and DEE.

The existence of an independent association between DEE and PPH was tested in multivariate logistic regression models. Because the data analyzed were hierarchical (level 1: women; level 2: maternity units), we used a multilevel logistic regression model with a random intercept. The covariates included in the final multivariate model were the individual and maternity units characteristics associated with DEE and with risk of PPH in the bivariate analysis. Because the source population came from a randomized trial, we also included a variable to indicate whether the unit had been randomized to the intervention or not. However, DEE was similar in the two randomization groups.

Clinically relevant interactions were examined, in particular those between DEE and each of the instrumental interventions, as well with oxytocin during labor.

We calculated the statistical power for our case-control study including 3,852 cases of PPH, 1,048 cases of severe PPH, and 762 controls. On the assumption that 10% were exposed to expulsive efforts longer than 37 minutes, the study had a 90% power to show an odds ratio (OR) of 1.5 or higher for the risk of PPH and of 76% for the risk of severe PPH.

The proportion of missing data for DEE was 19.3% in controls and 13.2% in cases. To take missing data into account in models, we chose to create a missing values category. The proportion of missing values was less than 5% for all other variables.

Statistical analyses were performed with Stata software, version 12.0.

## Results

Table 1 summarizes the characteristics of the case and control women. Cases were significantly older, had more often induced labor, received more oxytocin during labor, had more epidural analgesia, longer active stage of labor, more often instrumental delivery, episiotomy, perineal tear, higher birth weight babies and received less often prophylactic oxytocin. Cases delivered more often in large, public, and level 3 maternity units.

**Table 1:** Characteristics of cases and controls.

	Controls N = 762 n(%)	PPH N = 3852 n(%)	p	Severe PPH N = 1048 n(%)	p
**Maternal age (years)**					
≤25	187 (24.5)	943 (24.5)		243 (23.2)	
26–35	521 (68.4)	2536 (65.9)		709 (67.7)	
>35	54 (7.1)	369 (9.6)	0.09	96 (9.2)	0.26
**BMI (kg/m** ^**2**^ **)**					
≤25	545 (71.5)	2791 (72.5)		758 (72.3)	
26–30	79 (10.4)	467 (12.1)		121 (11.5)	
>30	28 (3.7)	193 (5.0)		53 (5.1)	
Missing value	110 (14.4)	401 (10.4)	0.22	116 (11.1)	0.38
**Gestational age (weeks)**					
<37	32 (4.2)	117 (3.1)		33 (3.1)	
37–40	594 (78.0)	2778 (74.5)		745 (71.1)	
≥41	136 (17.8)	953 (25.5)	<0.01	270 (25.7)	<0.01
**Induction of labor**	136 (17.9)	933(24.2)	<0.01	255 (24.3)	<0.01
**Total oxytocin dose during labor (IU)**					
0	213 (29.7)	797 (21.0)		203 (19.7)	
]0–0.57]	147 (20.5)	636 (16.8)		174 (16.9)	
]0.57–1.68]	179 (25.0)	926 (24.5)		227 (22.0)	
>1.68	178 (24.8)	1428 (37.7)	<0.01	428 (41.5)	<0.01
**Epidural**	647 (84.9)	3412 (88.6)	<0.01	920 (87.8)	0.08
**Duration of previous phases of labor (h) (mean +/- SD)**	5.3 ±2.8	6.1 ±3.1	<0.01	6.2 ±3.1	<0.01
**Mode of delivery**					
Spontaneous	565 (74.1)	2415 (62.7)		595 (56.8)	
Forceps	130 (17.1)	1124 (29.2)		373 (35.6)	
Vacuum	67 (8.8)	313 (8.1)	<0.01	80 (7.6)	<0.01
**Episiotomy**	433 (56.8)	2638 (68.5)	<0.01	773 (73.8)	<0.01
**Perineal tear**	209 (27.5)	1124 (29.2)	0.34	313 (29.9)	0.27
**Prophylactic postpartum oxytocin administration**	560 (73.5)	2270 (58.9)	<0.01	612 (58.4)	<0.01
**Birth weight (g)**					
≤3000	231 (30.3)	697 (18.1)		177 (16.9)	
3001–3500	332 (43.6)	1630 (42.3)		422 (40.3)	
3501–4000	176 (23.1)	1228 (31.9)		353 (33.7)	
>4000	22 (2.9)	293 (7.6)	<0.01	95 (9.1)	<0.01
**Annual number of deliveries in unit**					
≤1500	273 (35.8)	1284 (33.3)		345 (32.9)	
1501–3000	404 (53.0)	1980 (51.4)		524 (50.0)	
>3000	85 (11.2)	588 (15.3)	<0.01	179 (17.1)	<0.01
**Status of the maternity unit**					
Public university	162 (21.3)	1168 (30.3)		317 (30.2)	
Public non-university	374 (49.1)	1973 (51.2)		539 (51.4)	
Private	226 (29.7)	711 (18.5)	<0.01	192 (18.3)	<0.01
**Level of the maternity unit**					
Level 1	255 (33.5)	1095 (28.4)		293 (28.0)	
Level 2	390 (51.2)	1898 (49.3)		508 (48.5)	
Level 3	117 (15.4)	859 (22.3)	<0.01	247 (23.6)	<0.01
**Group of randomization in PITHAGORE6 trial**	396 (52.0)	1782 (46.3)	<0.01	529 (50.5)	0.53

Median DEE was 18 minutes (interquartile range: 10–27 minutes) among controls, 20 minutes (interquartile range: 14–30 minutes) among cases of PPH and 23 minutes (interquartile range: 15–32) among cases of severe PPH (p<0.01) (Table 2).

**Table 2:** Duration of expulsive efforts (EE) among cases and controls.

Duration of EE (minutes)	Controls N = 762	PPH N = 3852	p**	Severe PPH N = 1048	p**
**Median [interquartiles]**	18[10–27]	20 [14–30]	<0.01	23 [14–32]	<0.01
**Percentiles among controls (n (%)** *					
≤18 minutes	313 (50.9)	1307 (39.1)		316 (35.1)	
]18–27]	155 (25.2)	875 (26.2)		244 (27.1)	
]27–37]	88 (14.3)	654 (19.6)		185 (20.6)	
>37	59 (9.6)	508 (13.2)		155 (17.2)	
Missing	147 (19.3)	508 (13.2)	<0.01	148 (14.1)	<0.01

* For the missing data, percentages are calculated for the entire group. For the other categories, percentages are calculated in excluding the missing data

** Chi2 and Wilcoxon tests not taking missing data into account

The associations between DEE and risk of both PPH and severe PPH remained significant in multivariate analyses taking into account potential confounders.

With DEE considered as a continuous variable, the adjusted ORs for each additional 10 minutes of expulsive efforts were 1.11[1.02–1.21] for risk of PPH and 1.14[1.03–1.27] for risk of severe PPH.

When we considered DEE as a categorical variable, the adjusted OR for DEE >90^th^ percentile (i.e. >37minutes) was 1.39 [0.99–1.96] for the risk of PPH (reference: DEE≤18 minutes)(Table 3). Other factors significantly associated with an increased risk of PPH were total oxytocin dose higher than 1.68 IU, duration of active phase of labor, forceps delivery, episiotomy, perineal tears, and birth weight > 4000g. Factors significantly associated with a reduced risk of PPH were prophylactic postpartum oxytocin administration.

**Table 3:** Association between duration of expulsive efforts and risk of PPH–univariable and multivariable analyses (n = 4389; 3688 PPH and 701 controls).

Variables	crude OR OR[95% CI]	Adjusted OR* OR [95% CI]	p
**Duration of expulsive efforts (minutes)**			
≤18	1	1	
]18–27]	1.35 [1.10–1.67]	1.17 [0.92–1.48]	
]27–37]	1.78 [1.38–2.30]	1.32 [0.99–1.76]	
>37	2.06 [1.53–2.77]	1.39 [0.99–1.96]	
Missing	0.83 [0.66–1.03]	0.62 [0.47–0.83]	<0.01
**Maternal age**			
≤25	1.04 [0.86–1.24]	1.08 [0.88–1.33]	
26–35	1	1	
>35	1.40 [1.04–1.90]	1.29 [0.93–1.78]	0.27
**Gestational age**			
<37 weeks	0.78 [0.52–1.17]	1.17 [0.73–1.86]	
37–40	1	1	
≥41	1.50 [1.23–1.83]	1.11 [0.88–1.40]	0.56
**Induction of labor**	1.47 [1.21–1.80]	1.23 [0.96–1.56]	0.10
**Total dose of oxytocin (IU)**			
0	1	1	
]0–0.57]	1.16 [0.91–1.46]	1.16 [0.89–1.52]	
]0.57–1.68]	1.38 [1.11–1.72]	1.26 [0.97–1.64]	
>1.68	2.14 [1.73–2.66]	1.58 [1.19–2.10]	0.01
**Epidural**	1.38 [1.11–1.73]	0.85 [0.64–1.13]	0.27
**Duration of previous phases of labor (for one hour)**	1.10 [1.07–1.13]	1.04 [1.01–1.08]	0.01
**Mode of delivery**			
Spontaneous	1	1	
Forceps	2.02 [1.65–2.48]	1.58 [1.24–2.02]	
Vacuum	1.09 [0.83–1.45]	0.83 [0.59–1.76]	<0.01
**Episiotomy**	1.65 [1.41–1.94]	1.74 [1.38–2.18]	<0.01
**Perineal tears**	1.09 [0.92–1.30]	1.63 [1.29–2.06]	<0.01
**Prophylactic postpartum oxytocin administration**	0.52 [0.44–0.62]	0.48 [0.39–0.59]	<0.01
**Birth weight (grams)**			
≤3000	0.61 [0.51–0.74]	0.67 [0.54–0.84]	
3001–3500	1	1	
3501–4000	1.42 [1.17–1.73]	1.24 [0.99–1.54]	
>4000	2.71 [1.73–4.25]	2.23 [1.37–3.61]	<0.01
**Annual number of deliveries in the unit**			
≤1500	0.68 [0.52–0.88]	0.83 [0.46–1.50]	
1501–3000	0.71 [0.55–0.91]	0.80 [0.44–1.46]	
>3000	1	1	0.77
**Group of randomization in PITHAGORE6 trial**	0.80 [0.68–0.93]	0.89 [0.66–1.20]	0.45

* multilevel logistic regression model including all variables in the table

The risk of severe PPH was also significantly associated with DEE in the multivariate analysis, with an adjusted OR of 1.59 [1.06–2.39] for DEE >90^th^ percentile (i.e. 37minutes) (Table 4). Other factors significantly associated with an increased risk of severe PPH in the multivariate analysis were a total oxytocin total dose higher than 1.68 IU, active phase of labor, forceps delivery, episiotomy, perineal tears and birth weight > 4000g. The factors associated with a reduced risk of severe PPH were epidural analgesia and prophylactic postpartum oxytocin administration.

**Table 4:** Association between duration of expulsive efforts and risk of severe PPH–univariable and multivariable analysis (n = 1699; 998 severe PPH and 701 controls).

Variables	crude OR OR[95% CI]	Adjusted OR* OR[95% CI]	p
**Duration of expulsive efforts (minutes)**			
≤18	1	1	
]18–27]	1.56 [1.21–2.01]	1.37 [1.02–1.85]	
]27–37]	2.08 [1.55–2.81]	1.41 [0.99–2.02]	
>37	2.60 [1.86–3.65]	1.59 [1.06–2.39]	
Missing	1.00 [0.76–1.32]	0.62 [0.43–0.90]	<0.01
**Maternal age**			
≤25	0.95 [0.77–1.91]	1.08 [0.83–1.41]	
26–35	1	1	
>35	1.31 [1.22–1.52]	1.20 [0.80–1.81]	0.61
**Gestational age**			
<37 weeks	0.82 [0.50–1.35]	1.35 [0.73–2.51]	
37–40	1	1	
≥41	1.58 [1.25–2.00]	1.04 [0.78–1.40]	0.62
**Induction of labor**	1.49 [1.17–1.87]	1.15 [0.85–1.56]	0.36
**Total dose of oxytocin (IU)**			
0	1	1	
]0–0.57]	1.24 [0.93–1.66]	1.21 [0.85–1.72]	
]0.57–1.68]	1.33 [1.01–1.75]	1.23 [0.87–1.74]	
>1.68	2.52 [1.95–3.27]	1.82 [1.27–2.60]	<0.01
**Epidural**	1.28 [0.97–1.68]	0.67 [0.47–0.97]	0.03
**Duration of previous phases of labor (for one hour)**	1.12 [1.08–1.15]	1.04 [1.00–1.09]	0.05
**Mode of delivery**			
Spontaneous	1	1	
Forceps	2.72 [2.16–3.43]	1.98 [1.47–2.66]	
Vacuum	1.13 [0.80–1.60]	0.75 [0.49–1.15]	<0.01
**Episiotomy**	2.14 [1.75–2.61]	2.62 [1.91–3.59]	<0.01
**Perineal tears**	1.12 [0.91–1.38]	2.09 [1.53–2.86]	<0.01
**Prophylactic postpartum oxytocin administration**	0.51 [0.41–0.62]	0.44 [0.34–0.57]	<0.01
**Birth weight (grams)**			
≤3000	0.60 [0.47–0.77]	0.66 [0.49–0.89]	
3001–3500	1	1	
3501–4000	1.58 [1.25–1.99]	1.26 [0.96–1.65]	
>4000	3.40 [2.09–5.52]	2.80 [1.61–4.86]	<0.01
**Annual number of deliveries in the unit**			
≤1500	0.60 [0.44–0.81]	0.73 [0.40–1.34]	
1501–3000	0.62 [0.46–0.82]	0.73 [0.40–1.34]	
>3000	1	1	0.58
**Group of randomization in PITHAGORE6 trial**	0.94 [0.78–1.14]	0.91 [0.66–1.26]	0.58

* multilevel logistic model regression including all variables in the table

## Discussion

In this study, we found an independent association between the duration of expulsive efforts and the risk of PPH and severe PPH in nulliparas with a vaginal delivery. Interventions intended to reduce DEE, including oxytocin, episiotomy, and forceps delivery were also associated with an increased risk of PPH and severe PPH.

Specific data on the association between DEE and PPH are sparse with only 2 publications, both reporting a significant association[18, 19]. The first study, published in 2009, used data from the PEOPLE Canadian trial and reported an increased risk of PPH for a DEE between 2 and 3 hours (adjusted OR = 1.6 [1.0–2.5]) and between 3 and 4 hours (adjusted OR = 2.5 [1.54.1]), compared to a duration of less than 1 hour. In our study, we found a weaker association, but based on shorter durations. The different distribution of DEE in our study is due to very different obstetrical practices between Canada and France, where national recommendation is to intervene if delivery is not imminent after 30 minutes of expulsive efforts[18, 19, 22]. The second study, published in 2011 and based on data from the French PREMODA cohort, showed a risk of severe PPH (defined by blood loss >1000 mL and/or need for transfusion) associated with a DEE between 30 and 40 min (crude OR = 3.7 [1.2–11.7]) in comparison with DEE<10 min, a stronger[19]. However, in this study, authors assessed only the association between DEE and severe PPH, with a very small sample of severe PPH (n = 69) and could not take important confounding factors into account. In our population-based cohort-nested case control study from the PITHAGORE6 data, we were able to study a large sample of PPH and severe PPH with adjustment for the potential confounders associated with labor and delivery, which could not be studied in the previous two studies[18, 19]. For example, here, modeling the truncated duration of previous phases of labor provided us with a more accurate and realistic duration of the active phase of labor and, therefore, a more valid adjustment for this variable. Moreover, the precise quantitative data about the oxytocin doses administered allowed us to examine this important confounder[21].

Unlike the previous studies, ours was a population-based study, in which the characteristics of both the women and the maternity units were comparable to the national profile in the same time period, which enhances the external validity of our results.

Definition of PPH involved a physician’s or midwife’s judgment, based on a clinical estimate of blood loss. An underestimation of the number of PPH cases is thus possible, since clinicians tend to underestimate blood loss[23, 24]. Nonetheless, the addition of an objective criterion to identify PPH cases, i.e. a reduction in peripartum hemoglobin, enabled us to identify cases that were not found clinically. The definition of severe PPH was both objective and reproducible. The controls came from a random population sample representative of deliveries without hemorrhages. Accordingly, the definition and mode of selection of the two types of cases and the controls makes these comparative groups unlikely to be tainted by selection bias.

Our data come from a French trial. Thus, the extrapolation of our results is limited to countries with similar obstetrical practices. Indeed, French national guidelines recommend intervening if delivery is not imminent after 30 minutes of expulsive efforts, whereas US recommendations are to wait 3 hours of pushing among nulliparous women before diagnosing arrest of labor[14–16]. Durations of expulsive efforts observed in our study reflect French practices and can be considered very short for some obstetricians in other countries. The high number of cases with missing value for DEE is a limitation of our study. Women with missing values appeared to have a reduced risk of PPH. This greater proportion of missing DEE among controls most probably corresponds to medical files without any complications and therefore less thoroughly filled out and, likely, with shorter durations of expulsive efforts. It seems unlikely that the missing values might correspond mostly to instances with a long DEE. Estimates of the association between PPH and DEE were virtually the same when only complete cases were included in models, rather than a category for missing (data not shown). Finally, the variable “previous phases of labor” included duration at full dilatation before the beginning of expulsive efforts (i.e. passive 2^nd^ stage). The specific duration of this phase was not available in our study. Some women may have spent 1–2 hours or more at full dilatation before beginning to push. Thus, our results cannot be compared to studies evaluating consequences of duration of the second stage, i.e. the total duration at full dilatation before and during expulsive efforts.

The association between PPH and instrumental delivery has already been reported in the literature, in particular for the use of forceps[1, 25–28]. In addition, the use of forceps has been associated with a greater risk of PPH than vacuum extraction[29]. We did not see an increased risk of PPH associated with vacuum extraction, but we found a significant association between PPH and forceps. Nonetheless, we cannot exclude the possibility of an indication bias, where the choice between forceps or vacuum extraction is determined by the clinical situation. We thought that forceps is probably preferred in more complex situations, which are also at higher risk of PPH. Even though the association between PPH and vacuum extraction was not significant, our results certainly do not allow us to conclude that vacuum extraction would be a solution for reducing DEE.

To shorten the DEE, the primary available interventions are administration of oxytocin, episiotomy, or instrumental vaginal delivery. Accordingly, the dilemma of management of expulsive efforts persists, since, while a long DEE is associated with a higher risk of PPH, the risk is similar to that associated with the interventions intended to shorten this stage. It is therefore difficult to reach a conclusion about the best management strategy, in the absence of a randomized trial specifically designed to answer that question. Nonetheless, women's preferences and satisfaction must also be taken into account. For equal risks, it is likely that women prefer a spontaneous delivery, without episiotomy and with a longer period of pushing.

Finally, it is possible that repeated pushing leads to fatigue of the uterine muscle and thus also to a risk of atony in the immediate postpartum period. To reduce the duration of these expulsive efforts, it is necessary to push intensely. Accordingly, the association between expulsive efforts and PPH may be due more to the frequency of the pushing than to the duration of this stage itself, especially as the closer frequency of uterine contractions may be secondary to the use of progressively stronger doses of oxytocin intended to shorten expulsive efforts. Studies focusing specifically on the management of pushing and comparing intensive versus moderate management of expulsive efforts could be performed to answer this question. Such studies could also compare other outcomes, including children's well-being at birth and women's satisfaction. It is also possible that inefficient uterine action favors non progression of the fetus during the expulsive efforts, needing longer pushing efforts. In these situations, inefficient uterine action could persist after delivery, thus it could be an explanation for the uterine atony. This hypothesis should be tested by uterine electrophysiological studies.

## Conclusion

We found an independent association between the duration of expulsive efforts and the risk of PPH. However, beyond duration, other aspects of this phase may impact the risk of PPH. Defining the optimal management of expulsive efforts will require other studies, including randomized trials, in the future.
